# Validation of the Arabic coronavirus anxiety scale among mothers of infants in Saudi Arabia

**DOI:** 10.1186/s40748-026-00266-7

**Published:** 2026-07-03

**Authors:** Wid Kattan, Jennifer Barkin, Maryam Almutari, Lujain A. Khoja, Marwa Abdelmoaty, Khaled Alghamdi, Sulhi Ali Alfakeh

**Affiliations:** 1 Division of Psychiatry, Department of Medicine, College of Medicine, King Abdulaziz University, King Abdulaziz University Hospital, Jeddah, Saudi Arabia; 2 Mercer University School of Medicine, Georgia, USA; 3 King Fahad General Hospital, Jeddah, Saudi Arabia; 4 University of Jeddah, Jeddah, Saudi Arabia; 5 Ministry of Health, Riyadh, Saudi Arabia; 6 Ministry of Health, Jeddah, Saudi Arabia

## Abstract

**Objective:**

This research aims to verify the Arabic version of the Coronavirus Anxiety Scale (CAS) and to evaluate its association with maternal mental health among mothers of infants in the time of COVID-19 widespread in Saudi Arabia.

**Methods:**

A cross-sectional study was performed among the mothers of 1-month- to 1-year-old infants. Data collection was conducted between June and July 2020. Participants completed the Arabic CAS, Barkin Index of Maternal Functioning, Edinburgh Postnatal Depression Scale and Generalized Anxiety Disorder Scale. Descriptive statistics, Cronbach’s alpha, Pearson’s correlations and exploratory factor analysis (EFA), were applied.

**Results:**

Among 648 participants, the Arabic CAS showed better internal reliability. With the item loadings varying from 0.56 to 0.7, EFA validated a single-factor structure accounting 65.5% of overall variance. The Arabic CAS then demonstrated high internal consistency (Cronbach’s α = 0.89). CAS scores correlated negatively with Barkin Index of Maternal Functioning (BIMF) and positively with Edinburgh Postnatal Depression Scale (EPDS) and Generalized Anxiety Disorder-7 (GAD-7) supporting construct validity.

**Conclusion:**

The Arabic CAS shown adequate validity and reliability for evaluating COVID-19 linked anxiety among mothers of infants in Saudi Arabia. This tool can be used to detect the distress related to pandemic in postpartum individuals.

## Introduction

On March 11, 2020, a pandemic named COVID-19 across the world was proclaimed by the WHO, which was initially detected at Wuhan, China [1]. Within a short time, this severely infectious viral infection has swept across the countries, upending the healthcare systems, social structures and economy. On March 2, 2020, The Saudi Arabia government announced their first case and they quickly implemented the preventive measures like suspending international travel, obligatory use of mask, shut down of educational institutes, home quarantine, restricting the gatherings [2, 3]. These public health interventions, despite the necessity caused the psychological problems among the population due to change in lifestyle, social isolation and fear of infection [4].

Global health pandemics tend to cause the extensive fear and uncertainty, that can worsen the anxiety and stress-related disorders in individuals [5, 6]. In contrast to the earlier infectious outbreaks, the COVID-19 pandemic was for longer time and characterized by an extensive sense of unpredictability, resulting to psychological repercussion. Studies undertaken during and later the pandemic consistently shown the higher rates of generalized anxiety, depressive symptoms, sleeplessness and post-traumatic stress [7–10]. WHO in 2021 projected a 25.0% hike in occurrence of depression or anxiety disorders, highlighting the mental health burden caused by pandemic [11].

Nevertheless, psychological responses to the pandemic were not same across different demographics. Some demographic and psychosocial categories mainly women, residents in urban and people with chronic illness or with psychiatric disorders exhibited increased vulnerability [12, 13]. Among these categories, postpartum women are uniquely vulnerable population, given the hormonal, psychosocial and physiological changes occur during the perinatal phase. Evidence shows that the amount of depressive and maternal anxiety were worsen in the time of pandemic due to concerns like fear of infection, limited healthcare services, insufficient social support, and uncertainty about the newborns safety [14–17]. The amalgamation of pandemic stress and postpartum vulnerability have increased the chances of postpartum anxiety and depression, which could potentially affect maternal–infant bonding and long-term developmental outcomes for child [18–20].

Although there are numerous instruments (e.g., GAD-7, EPDS) available to measure generalized depression or anxiety, these scales fail to capture anxiety related to contagious disease threats such as COVID-19. To fill these gaps, Coronavirus Anxiety Scale was created to study stomatic based anxiety symptoms that are linked to fear related to coronavirus [21]. This CAS having five items reflects somatic symptoms of anxiety—insomnia, loss of appetite, dizziness, tonic immobility, and gastrointestinal distress—scaled on a 5-point Likert scale. The instrument demonstrated robust psychometric properties in multiple populations, with a cutoff score of nine differentiating functional from dysfunctional anxiety [22].

Since its development, the CAS has been interpreted and validated across multiple languages consisting Bangla, Korean, Turkish, Polish, Urdu, and Brazilian Portuguese [23–28]. An Arabic translation was also reported, tested among healthcare workers and general adult populations in Saudi Arabia, showing acceptable reliability and validity [29, 30]. However, no validation studies have concentrated on Arabic-speaking postpartum women yet, who represent a demographically and psychologically distinct subgroup. The postpartum period involves biological fluctuations and heightened emotional sensitivity that may alter the psychometric structure of anxiety measures compared to the general population. Hence, direct adoption of the general population Arabic version of the CAS may not ensure valid measurement among mothers in early motherhood.

CAS among postpartum women is of particularly considered because they represent a specially vulnerable category due to hormonal, physiological, and psychosocial changes, which may impact how anxiety—mainly stress-related, somatic symptoms—is expressed and experienced. They may encounter additional stressors, including concerns about infant health, disruptions to healthcare availability and decreased social support. These elements may change the manifestation of anxiety symptoms, making it crucial to evaluate whether a tool like the CAS, which concentrates on physiological anxiety responses to pandemic-related stress, shows adequate validity and reliability.

Although EPDS and GAD-7 are widely used in postpartum mental-health research, they assess generalized depressive and anxiety symptoms and do not capture the somatic fear responses triggered by infectious-disease threats. The CAS was specifically developed to measure physiological anxiety reactions—such as dizziness, tonic immobility, gastrointestinal distress, and sleep disturbance—elicited by COVID-19-related cues.

Postpartum women may experience heightened pandemic-specific fears, including concerns about infecting their newborn, reduced access to healthcare, and restrictions on support persons during delivery. These fears often manifest physiologically, making the CAS uniquely suited to detect pandemic-related anxiety in this population. Validating the Arabic CAS in postpartum women therefore addresses a conceptual and clinical gap not covered by existing tools.

The present study therefore focuses to validate the Arabic version of the CAS among mothers of infants in Saudi Arabia, a group particularly vulnerable to pandemic-induced psychological distress. By studying the factor structure, convergent validity and internal consistency in this specific cohort, we sought to determine whether the CAS adequately captures coronavirus-related anxiety in postpartum contexts. Even though comparable validations were previously conducted in general or healthcare individuals, this research offers the new evidence on the structural stability and applicability of the CAS in postpartum women.

## Materials and methods

### Methodology

#### Study design and participants

This method is component of a broader research investigating maternal functioning and mental health indicators in the COVID-19 in Saudi Arabia. The main goal of this segment was to verify the Arabic version of CAS in postpartum women. This cross-sectional, community-oriented investigation involved mothers of live-born infants aged between 1 month to 1 year. A convenience sampling method was adopted, using E-survey through links the data was collected. The data was gathered during June–July 2020, which is the pandemic’s peak period for comprehending the long-term psychosocial sequelae and guiding preparedness for future public health pandemics.

##### Eligibility criteria

Participants were considered only if they

A. Were Saudi Arabia residing mothers.
B. Have a living infant aged between 1 month to 1 year.
C. Were willing and able to do the Arabic online questionnaire.

##### Sample size

At least 5–10 participants for item were suggested as cited in many earlier studies. With 5 CAS items, 648 participants were assessed to ensure sufficient statistical power for psychometric evaluation.

### Measures

#### Coronavirus anxiety scale

This is an instrument consisting 5-items for assessing stomatic-related anxiety particularly related to COVID-19 during the last two weeks, consisting insomnia, loss of appetite, tonic immobility, and gastrointestinal distress. Likert scale with 5 points varying from 0 = not at all to 4 = daily used to rate items. The scale was originally developed and validated by Lee (2020). Score and anxiety are directly proportional. Accordance to the ISPOR guidelines, the Arabic version was translated and culturally adjusted.

#### Maternal functioning

The Barkin Index of Maternal Functioning is a self-report instrument consisting 20-items valued on a Likert scale with 7 points varying 0 = firmly contradict to 6 = firmly accept. Total score vary from 0 indicating as lowest functioning to 120 as highest functioning. The original validation was reported by Barkin et al. (2010). The Arabic version has been validated (Cronbach’s α = 0.87) earlier.

#### Anxiety

The Generalized Anxiety Disorder-7 comprises 7-item scale evaluating general anxiety for the last two weeks. Every item is valued on a Likert scale with 4 points: 0 represents firmly not to 3 as almost daily. Scores > 5, >10, and > 15 means meek, medium and extreme anxiety. The tool was developed and validated by Spitzer et al. (2006). The Arabic version has shown satisfactory reliability (Cronbach’s α = 0.763), as reported in regional validation studies Sawaya et al., (2016).

#### Depressive symptoms

The Edinburgh Postnatal Depression Scale is made up of 10 items evaluating mood and depressive symptoms experienced in the previous week, consisting self-harming thoughts, sadness, panic, worrying, and difficulties in sleeping. Every item is valued on a scale with 4 points. Scores ≥ 10 represents possible depression, and scores ≥ 13 represents probable depressive illness. The EPDS was originally developed by Cox et al. (1987). The Arabic version has been validated in earlier works Ghubash et al., (1997).

### Data collection procedure

The survey was administered using the Google Forms platform, which automatically timestamps entries and restricts multiple submissions from the same device. The survey link was distributed publicly through Twitter, WhatsApp, and Facebook. Participation was voluntary, and no incentives were offered.

Before accessing the questionnaire, participants viewed a mandatory electronic informed-consent page describing study aims, confidentiality, data handling, and the right to withdraw at any time. Only participants who selected ‘I agree’ were allowed to proceed.

All survey items were set as ‘required,’ preventing missing data. Google Forms’ built-in ‘limit to one response per device’ feature was activated to reduce duplicate entries. No IP addresses, emails, or personal identifiers were collected. Data were stored on encrypted institutional Google Workspace servers accessible only to the research team.

Because the survey link was distributed through open social media channels, the number of individuals who viewed the invitation could not be determined; therefore, a traditional response rate could not be calculated.

In accordance with the CHERRIES checklist, the following elements are reported: – Design: Cross-sectional online survey. – IRB approval: King Abdulaziz University Hospital (No. 339 − 20). – Survey platform: Google Forms. – Recruitment: Public social media posts; no targeted advertising. – Consent: Mandatory electronic consent page. – Duplicate prevention: ‘Limit to one response per device’ enabled. – Data completeness: All items required; no missing data. – Incentives: None. – Response rate: Not calculable due to open-access distribution. – Data security: Encrypted institutional Google Workspace storage. – Analysis: Only complete submissions included.

Individuals with highest scores on EPDS or GAD-7 were the details on official free mental health hotlines and online counseling services in Saudi Arabia. No direct assistance was offered by the research team.

### Statistical analysis

All statistical analysis were performed with IBM SPSS Statistics.

#### Descriptive statistics

Continuous variables are represented as average± standard deviation, whereas categorical variables was represented as percentages and counts. Data normality was evaluated using the Shapiro–Wilk test and visual inspection of histograms. For non-normally distributed data, variables were summarized using median and interquartile range.

#### Reliability

Internal consistency was evaluated using McDonald’s ω and Cronbach’s α, along with corrected item–total correlations, in which each item was correlated with the total scale score excluding that item. Pearson’s correlation coefficients were used for these evaluations.

#### Factor analysis

Exploratory factor analysis was performed to study factor loadings, communalities, eigenvalues, and total variance described. Confirmatory factor analysis (CFA) evaluated model suitable with the help of χ²/df, CFI, RMSEA, SRMR and TLI.

#### Construct validity

Pearson’s correlations were determined between GAD-7 and CAS, BIMF-20 and EPDS scores to evaluate convergent and discriminant validity.

### Ethical consideration

King Abdulaziz University Hospital’s review board permitted the ethical consent (Approval No. 339 − 20). There was minimal risk in participation. Informed consent was acquired and throughout the survey confidentiality was kept. Participants requiring psychological support were referred to existing free mental health services.

## Results

For the evaluation of the study a total of 648 participants who had recently given birth were considered. The average age of mothers was 32.1 years (SD = 5.3) [Median = 32.0, range = 21 to 50]. Participants children had a mean age of 5.8 months (SD = 3.9) [Median = 5.0, range = 1 to 12]. Participants had an average of 2.0 children (range = 1 to 8). The mean total scores were BIMF-20 = 85.6 (SD = 15.3), EPDS = 12.3 (SD = 5.3), CAS = 2.0 (SD = 3.3), and GAD-7 = 8.2 (SD = 5.2).

The most of participants have a university degree qualification (65.4%), underwent natural deliveries (62.3%), residents as Saudi (92.0%), were married (99.4%), and stated no prior psychiatric problems (91.0%). A portion of family members had not tested positive for COVID-19 (79.3%), had not infected positive themselves (95.8%), and shown that the pandemic did not severely impact their childbirth (79.3%) or postpartum experiences (59.4%). Moreover, 67.6% were allowed to have a family member available during the delivery, and 64.5% felt they obtained sufficient social support after delivery (Table 1).

**Table 1:** Socio-demographic characteristics of the patients who completed the coronavirus anxiety scale (CAS) (*N* = 648). Gives a summary of the socio-demographic, postpartum experiences, COVID-19 related experiences of the participants and and mean scores of depression, maternal infant bonding and anxiety scales

Baseline Characteristics		% (*n*)
Nationality		
Saudi		92.0(596)
Non-Saudi		8.0(52)
Educational level		
Elementary school		0.3(2)
Middle school		0.9(6)
High school		6.6(43)
University education		65.4(424)
High education		26.7(173)
Employment status		
Unemployed		46.3(300)
Postpartum employed (you have career plans nearby or are on the job right now)		7.9(51)
Employed from birth		45.8(297)
Average monthly income for the family		
Less than 5,000 SAR		7.6(49)
$\:\mathrm{5,000}-\mathrm{10,000}$ SAR		20.7(134)
10,001–15,000 SAR		21.8(141)
15,001–20,000 SAR		13.9(90)
20,001–25,000 SAR		8.6(56)
25,001–30,000 SAR		7.3(47)
More than 30,000 SAR		13.7(89)
Unknown		6.5(42)
Marital Status		
Married		99.4(644)
Divorced		0.6(4)
Birth type		
Natural		62.3(404)
CS		37.7(244)
Past psychiatric history		
Yes		9.0(58)
No		91.0(590)
If you were tested for the Coronavirus, did the result appear positive?		
Yes		4.2(27)
No		95.8(621)
Have any of your family members or those in contact with you been diagnosed with the Coronavirus?		
Yes		20.7(134)
No		79.3(514)
Has the Covid-19 pandemic negatively affected your childbirth experience?		
Yes		20.7(134)
No		79.3(514)
Has the Covid-19 pandemic negatively affected your postpartum experience?		
No		59.4(385)
Were you allowed to take a family member with you during the birth?		
Yes		67.6(438)
No		32.4(210)
Did you feel you have got the social support you need in the postpartum period?		
Yes		64.5(418)
No		35.5(230)
	Mean (SD)	Median (range)
Age	32.1(5.3)	32.0(21–50)
The age of your child (infant)	5.8(3.9)	5.0(1–12)
How many children do you have	2.4(1.5)	2.0(1–8)
BMIF Factor 1–9 items	44.4(6.3)	45.0(12–54)
BMIF Factor 2–4 items	16.3(5.0)	17.0(0–24)
BMIF Factor 3 − 2 items	6.7(3.1)	7.0(0–12)
BMIF Factor 4–8 items	30.9(8.2)	32.2(0–48)
BMIF-20	85.6(15.3)	87.0(32–120)
Edinburgh Perinatal/Postnatal Depression Scale	12.3(5.3)	12.0(1–30)
Coronavirus Anxiety Scale	2.0(3.3)	1.0(0–20)
GAD-7 Anxiety	8.2(5.2)	7.0(0–21)

Table 2 provides the complied descriptive statistics for the CAS. Participants exhibited low levels of anxiety associated to the coronavirus, as revealed by an average total CAS score of 2.0 (SD = 3.3). Over 70% of respondents have a mean score for individual items ranging from 0.3 to 0.6, reporting they “never” experienced anxiety symptoms when revealed to coronavirus-linked information. This trend was constant for the items linked symptoms like freezing or paralysis, appetite loss, ache in stomach, issue related to sleeping and feeling dizzy. These lesser mean scores depicts a floor effect, showing that majority of mothers felt lesser anxiety associated to the coronavirus.

**Table 2:** Coronavirus Anxiety Scale (CAS) distribution of the studied population (*N* = 648). Represents the frequency and percentage distribution of participants responses to each item of the CAS, indicating the anxiety related symptoms experienced and reflecting overall levels of maternal functioning

Item	Strongly disagree	Disagree	Somewhat t disagree	Neutra I	Somewhat t agree	Agree	Strongly y agree	Mean (SD)
	(0)	(1)	(2)	(3)	(4)	(5)	(6)	
	%	%	%	%	%	%	%	
1	0.9	1.1	2.9	4.8	22.2	42.4	25.6	4.8(1.1)
2	7.9	16.2	17.7	10.0	29.6	15.4	3.1	3.0(1.7)
3	2.8	3.9	6.3	5.4	13.1	27.2	41.4	4.7(1.6)
4	1.2	2.0	5.6	6.2	26.1	38.0	21.0	4.5(1.3)
5	2.2	5.2	9.7	9.9	32.4	30.2	10.3	4.0(1.4)
6	6.5	6.6	6.0	4.2	21.1	27.5	28.1	4.2(1.8)
7	3.2	3.7	5.6	5.1	21.1	33.0	28.2	4.5(1.5)
8	5.2	8.3	9.3	10.5	21.8	33.3	11.6	3.8(1.7)
9	4.3	8.3	9.0	14.4	23.3	28.5	12.2	3.8(1.6)
10	0.5	0.9	0.9	3.5	9.1	32.3	52.8	5.3(1.0)
11	8.6	14.5	12.0	15.1	19.1	18.2	12.3	3.3(1.9)
12	0.5	0.0	0.6	0.6	5.4	29.2	63.7	5.5(0.8)
13	3.7	4.0	7.9	4.8	24.2	31.5	23.9	4.3(1.6)
14	0.5	0.2	0.5	2.5	12.5	38.4	45.5	5.2(0.9)
15	1.2	4.8	5.4	7.6	27.8	33.3	19.9	4.4(1.4)
*16	5.6	8.8	10.2	8.3	10.8	27.2	29.2	4.1(1.9)
17	3.7	6.9	11.9	9.4	33.3	26.4	8.3	3.7(1.5)
*18	14.2	17.9	23.0	13.0	10.5	15.9	5.6	2.6(1.8)
19	0.6	0.5	0.5	2.8	14.8	45.1	35.8	5.1(0.9)
20	0.8	1.9	2.2	3.9	14.5	42.6	34.3	4.9(1.2)

*Note. Response coding for Items 16 and 18 is as follows: Strongly Disagree = 6; Disagree = 5; Somewhat Disagree $\:=4$; Neutral $\:=3$; Somewhat Agree $\:=2$; Agree $\:=1$; Strongly Agree $\:=0$

The CAS showed internal consistency (Cronbach’s α = 0.89) than previous studies. The corrected item-total correlations varied from 0.61 to 0.77, indicating sufficient item similarity. When individual items were not considered, Cronbach’s alpha values obtained ranging 0.85–0.88 indicating that no individual item substantially affected the internal consistency of the scale, verifying that all items positively influenced to the scale’s overall reliability as seen in Table 3.

**Table 3:** Mean frequency distribution of responses to Coronavirus Anxiety Scale (CAS) Items (*N* = 648). Presents the mean frequency distribution of responses to the CAS items among 648 participants, representing that many respondents reported “never” experiencing anxiety symptoms, with low mean scores across all items

Item	Never	Rarely for less than a day or two	For several days	For more than 7 days	Almost every day	Mean (SD)
	(0)	(1)	(2)	(3)	(4)	
	%	%	%	%	%	
1	74.5	12.8	9.1	1.5	2.0	0.4(0.9)
2	61.7	23.0	9.1	3.7	2.5	0.6(1.0)
3	78.1	13.9	5.6	1.2	1.2	0.3(0.7)
4	83.5	8.8	4.9	1.7	1.1	0.3(0.7)
5	76.2	15.7	5.7	1.5	0.8	0.3(0.7)

The inter‑item correlations of the Coronavirus Anxiety Scale (CAS) demonstrated consistently strong associations across all five items. As shown in Table 4, correlations ranged from 0.48 to 0.70, with the highest association observed between Items 4 and 5 (“0.70”) and the lowest between Items 1 and 4 (“0.48”). These values indicate solid internal coherence among the items and support the scale’s unidimensional structure.

**Table 4:** Coronavirus Anxiety Scale (CAS) Inter-item correlations (*N* = 648). Represents the inter-item correlations of the CAS, which put forwards good internal consistency of the scale

Item	$\:1$	$\:2$	$\:3$	$\:4$	$\:5$
1	1.0	0.55	0.54	0.48	0.51
2		1.0	0.54	0.52	0.54
3			1.0	0.67	0.63
4				1.0	0.70
5					1.0

Exploratory factor analysis further confirmed the adequacy of the data for factor extraction. Sampling adequacy was high (KMO = 0.87), and Bartlett’s test of sphericity was significant (χ² = 1025.4, *p* < 0.001), indicating that the correlation matrix was factorable. Principal‑component extraction with varimax rotation yielded a single factor with an eigenvalue of 3.27, accounting for 65.5% of the total variance. All items demonstrated strong factor loadings (> 0.70) and communalities ranging from 0.56 to 0.71, confirming the robust one‑factor structure of the CAS. As all items met the retention criteria (loading ≥ 0.40), no items were removed.

Confirmatory factor analysis (CFA) was conducted using maximum-likelihood estimation to further evaluate the construct validity of the Coronavirus Anxiety Scale (CAS). As shown in Table 5, all items demonstrated strong standardized factor loadings ranging from 0.75 to 0.84, consistent with a robust single-factor structure. The one-factor model exhibited satisfactory fit indices (χ²/df = 2.38, TLI = 0.93, RMSEA = 0.045, CFI = 0.95, SRMR = 0.032), indicating that a single latent factor adequately represents coronavirus-related anxiety. These findings reinforce the unidimensionality suggested by the exploratory factor analysis and confirm that all items contribute meaningfully to the underlying construct.

**Table 5:** Factor Loadings for the Coronavirus Anxiety Scale (CAS) (*N* = 648). Presents the factor loadings of the CAS items, indicating strong loadings on a single factor and an eigenvalue of 3.27

Item	CAS
1	0.75
2	0.77
3	0.84
4	0.84
5	0.84
Eigenvalues	3.27

The outcomes of the discriminant and concurrent validity are presented in Table 6. By the BIMF-20 total score, the total CAS score revealed a notable negative correlation, suggesting that greater maternal functioning was associated with lower levels of COVID-19 related anxiety.

**Table 6:** Concurrent and Discriminant Validity for the Coronavirus Anxiety Scale (CAS) (*N* = 648). Indicates that the CAS shows an important negative correlation with BIMF-20 and positive correlations with anxiety and depression measures, supporting its concurrent and discriminant validity

Parameter	Coronavirus Anxiety Scale
BIMF-20	
r	-0.229
p-value	< 0.001
N	648
Edinburgh Perinatal/Postnatal Depression Scale	
r	0.404
p-value	< 0.001
N	648
GAD-7 Anxiety	
r	0.388
p -value	< 0.001
N	648

Table 6 presents the results for concurrent and discriminant validity. With BIMF-20 total score, it represented a notable negative correlation implying that higher maternal functioning was associated by lesser levels of coronavirus-linked anxiety. By the GAD-7 and EPDS scores positive correlations were seen, proposing higher anxiety due to covid is caused due to increase in symptoms. The outcomes of the correlations suggests moderate convergent validity, validating that the CAS measures are related but definite construct.

Table 7 represents the outline of the variance detailed for the CAS items by principal component analysis.

**Table 7:** Total Variance Explained of CAS items using principal component analysis as an extraction method (*N* = 648). Shows that the Coronavirus Anxiety Scale items have strong communalities and collectively explain 65.48% of the total variance, representing a solid factor structure using principal component analysis

Communalities	Extraction
CAS1	0.564
CAS2	0.596
CAS3	0.704
CAS4	0.702
CAS5	0.708
Total Variance Explained	$\:65.48\mathrm{\%}$

Extraction Method: Principal Component Analysis

The outline of the variance detailed for the CAS items is presented in Table 7. The cumulative explained variance = 65.48%, with extraction values ranging 0.564–0.708. These results indicate that the CAS successfully captures the variance in coronavirus-related anxiety symptoms among postpartum women, with the most contributions from items associated with nausea or cramping, while the least from items related to dizziness or light-headedness.

## Discussion

The present research focuses to verify the Arabic version of the CAS among Saudi Arabian postpartum females. Unlike general anxiety or depression scales, the CAS was mainly created to study stomatic-lined anxiety symptoms directly linked to the COVID-19, consisting disturbance in sleeping, loss in appetite, dizziness, tonic immobility, and gastrointestinal distress [24]. This focus permits for a more accurate evaluation of pandemic-linked anxiety in populations mostly vulnerable to stress, such as mothers during the postpartum period. Postpartum women experience major physiological, hormonal, and psychosocial changes, making it crucial to assess whether the mechanisms such as CAS carry reliability and validity in this subgroup, other than considering psychometric similarity with general adults.

Our findings illustrated that the Arabic CAS exhibit allowable internal consistency in postpartum women, along with Cronbach’s alpha values ranging with the earlier studies in other populations [25–30]. Factor analysis validated the unidimensional structure of the scale, with all items representing reasonable factor loadings and communalities, and a total variance of over 65% was explained. This level of variance is consistent with earlier validation studies of the CAS across different populations. In psychometric research, a variance of 50% or greater is usually considered acceptable for a unidimensional construct, representing that the extracted factor sufficiently represents the underlying concept. Therefore, the variance observed in this evaluation backs the robustness of the CAS in this population. These results show that the CAS dependably captures coronavirus-linked anxiety in this particular cohort. Moreover, CAS scores were positively corresponds with both the GAD-7 and the EPDS, proposing a better convergent validity, and negatively correlated with maternal ability as evaluated by the BIMF-20, depicting expected discriminant associations. These results assist the CAS as a psychometrically sound tool for studying pandemic-specific anxiety in postpartum mothers.

Although EFA and CFA were conducted on the same dataset, this approach is acceptable in scale validation when external datasets are not available, particularly when the factor structure is theoretically well‑established and the sample size is large. The CAS has repeatedly demonstrated a stable one‑factor structure across multiple populations and languages, reducing the risk of model overfitting.

The average CAS scores in our sample were relatively less, a result that may be affected by various factors. Initially, the timing of data gathering was performed in June and July 2020, aligned with the primary stages of pandemic management in Saudi Arabia, when strict preventive measures and awareness campaigns may have eased immediate anxiety responses. Secondly, cultural and social factors, consisting stigma related with mental health reporting, may have helped to lessen the self-reported anxiety. Thirdly, some CAS items, like gastrointestinal distress or tonic immobility, might not totally affect postpartum-specific anxiety manifestations, possibly impacting the total scoring. While performing CAS in postpartum mothers, these views highlights the requirement of contextual interpretation.

In contrast to general depression or anxiety measures, CAS provides a unique function. Where EPDS and GAD-7 studies concentrated on generalized depressive and anxiety features, the CAS mainly aims coronavirus-linked anxiety, identifying stomatic-related responses to pandemic-linked hazards. This variation accounts its use even while mean anxiety scores are lesser, as it is able to recognize participants having dysfunctional anxiety that might not be shown in traditional scales. Moreover, the CAS can align with the available measures by giving particularly aimed insights into pandemic-specific stressors, providing psychosocial guidance whenever required.

Furthermore, the CAS offers conceptual advantages over EPDS and GAD-7 in the context of infectious-disease anxiety. While EPDS and GAD-7 assess general depressive and anxiety symptoms, the CAS captures somatic fear responses specifically triggered by COVID-19-related cues. These physiological reactions—such as dizziness, gastrointestinal distress, and tonic immobility—are not assessed by traditional postpartum mental-health tools. Given the unique pandemic-related fears experienced by postpartum women, the CAS provides complementary and clinically relevant information.

The findings support the Arabic CAS as a valid and reliable tool for evaluating COVID-19 related anxiety among postpartum mothers in Saudi Arabia. The scale explained satisfactory internal consistency, a stable factor structure, and expected correlations with related mental health measures. However, interpretation of CAS scores should consider temporal, cultural, and postpartum-specific factors. Future research should further assess its applicability across wider populations, considering longitudinal and test–retest evaluations.

## Conclusion

The study demonstrates that the Arabic CAS is a valid and reliable instrument for evaluating COVID-19–related anxiety among mothers of infants in Saudi Arabia. The scale showed high internal consistency (Cronbach’s α = 0.89), a unidimensional factor structure describing 65.5% of the total variance, and correlations with related measures, including the EPDS and GAD-7, backing its construct validity. These outcomes represent that the Arabic CAS can help as an effective screening tool for identifying pandemic-related anxiety in postpartum mothers. Given their increased psychological vulnerability during the postpartum period, this tool may assist early detection and support relevant clinical and research applications. Further studies are recommended to assess its performance in wider populations and across various time points.

## Limitations and Recommendations

This research has certain constraints. Its cross-sectional approach assesses maternal anxiety and mental health variables at one specific moment, which restricts causal inference. The dependence on self-reported surveys may lead to recall or social desirability bias, which can impact the reliability of answers. The sample is limited to mothers with infants aged 1 to 12 months from selected areas in Saudi Arabia, potentially restricting the applicability to other populations. Furthermore, the lack of structured clinical interviews limits the precision of diagnoses, and the context specific to the pandemic may have affected anxiety levels, indicating a need for additional validation of the Arabic CAS outside of COVID-19 conditions.

The research also points procedural considerations and drawbacks. Using social media the data was gathered, it shows possible selection favoritism and limits the ability to confirm respondents authenticity. Following the privacy and consent steps the online method may favor mothers having digital knowledge and access to internet probably restricting generalizability. Moreover, this research only depend on self-assessment measures and doesn’t have test-retest reliability evaluations, that might later reinforce psychometric assessment. Broader recruitment approaches and multiple time points should be included in the later studies to improve the outcomes. And also the participants were bounded to mothers of infants which may not speak of postpartum mothers in later stages or women from the other population. Moreover, this study depended on self-reported measures, which may cause response bias. Particularly, self-desirability bias may have impacted participants’ responses, as they may underreport or overreport symptoms to align with perceived cultural or social expectations.

Future studies should involve longitudinal and test-retest evaluations to examine the consistency and predictive validity of the Arabic CAS throughout various postpartum phases and broaden its validation to include a wider range of Arabic-speaking populations, such as expectant mothers and fathers. Integrating the scale into regular maternal health assessments can aid in the early identification and intervention of anxiety during health emergencies. Comparative studies across multiple countries are suggested to establish its standardization in different contexts. In summary, the Arabic CAS shows promise for implementation within clinical and public health environments in Saudi Arabia to enhance maternal mental health during and after pandemics.

## Data Availability

Without undue reservation, the authors will make available of the raw data backing the conclusions of this article.
